# Learning from complaints in healthcare: a realist review of academic literature, policy evidence and front-line insights

**DOI:** 10.1136/bmjqs-2019-009704

**Published:** 2020-02-04

**Authors:** Jackie van Dael, Tom W Reader, Alex Gillespie, Ana Luisa Neves, Ara Darzi, Erik K Mayer

**Affiliations:** 1 Centre for Health Policy, Institute of Global Health Innovation, Imperial College London, London, UK; 2 Department of Psychological and Behavioural Science, London School of Economics and Political Science, London, UK

**Keywords:** health policy, patient-centred care, patient safety, adverse events, epidemiology and detection, governance

## Abstract

**Introduction:**

A global rise in patient complaints has been accompanied by growing research to effectively analyse complaints for safer, more patient-centric care. Most patients and families complain to improve the quality of healthcare, yet progress has been complicated by a system primarily designed for case-by-case complaint handling.

**Aim:**

To understand how to effectively integrate patient-centric complaint handling with quality monitoring and improvement.

**Method:**

Literature screening and patient codesign shaped the review’s aim in the first stage of this three-stage review. Ten sources were searched including academic databases and policy archives. In the second stage, 13 front-line experts were interviewed to develop initial practice-based programme theory. In the third stage, evidence identified in the first stage was appraised based on rigour and relevance, and selected to refine programme theory focusing on what works, why and under what circumstances.

**Results:**

A total of 74 academic and 10 policy sources were included. The review identified 12 mechanisms to achieve: patient-centric complaint handling and system-wide quality improvement. The complaint handling pathway includes (1) access of information; (2) collaboration with support and advocacy services; (3) staff attitude and signposting; (4) bespoke responding; and (5) public accountability. The improvement pathway includes (6) a reliable coding taxonomy; (7) standardised training and guidelines; (8) a centralised informatics system; (9) appropriate data sampling; (10) mixed-methods spotlight analysis; (11) board priorities and leadership; and (12) just culture.

**Discussion:**

If healthcare settings are better supported to report, analyse and use complaints data in a standardised manner, complaints could impact on care quality in important ways. This review has established a range of evidence-based, short-term recommendations to achieve this.

## Introduction

A steady rise in patient complaints has been accompanied by increasing efforts to effectively analyse complaints for quality improvement. In England’s National Health Service (NHS), the number of formal complaints received yearly has doubled to over 200 000 between 2008 and 2018.^1 2^ Complaints are complex narratives that report on perceived failures of healthcare delivery from the patient’s perspective. Complaints have been recognised as a valuable source of data for a number of reasons. Unlike most patient feedback mechanisms (eg, patient satisfaction surveys, patient consultations), complaints are unsolicited: they represent the care issues that breach a threshold of concern and compel patients and families to take action. This includes safety incidents^3–6^ and poor experiences^7–9^ that are not always identified in internal systems of healthcare monitoring (eg, incident reports, retrospective case reviews). Complaints contain data on difficult-to-monitor areas of practice,^10^ such as care access or continuity, systemic problems and care omissions. Complaints further describe clinical, social and institutional aspects of perceived care failures^5 11 12^; capturing sociostructural, or ‘systems’,^13 14^ dimensions to error and negligence.^15^ However, in contrast to standard feedback and incident reporting mechanisms, complaints systems are not primarily designed for quality monitoring and improvement, and predominantly concern processes to provide individual complainants with a formal response; that is, complaint handling (box 1).^16 17^

Box 1 **Definition of terms used in this review**

**Complaint terminology**

*Complaint:* ‘a formal communication reporting a failure that seeks an institutional response’^10^

*Complaint handling:* receiving and responding to individual complainants, typically performed by a complaints department

*Quality monitoring and improvement:* standardised reporting and aggregated analysis of complaints data to generate continuous improvement insights at an organisational and national level

*Patient-centric responding:* the institution’s communication to individual complainants in response to their complaint, including response elements important to complainants (eg, an explanation of poor care, expression of responsibility, learning or action taken)

**Realist review terminology**

*Programme theory*: the ‘underlying assumptions about how an intervention is supposed to work’^32^

*Contexts:* ‘aspects of the background, people and setting that moderate outcomes’^114^

*Mechanisms:* ‘underlying entities, processes, or structures which operate in particular contexts to generate outcomes of interest’^115^

*Outcomes:* ‘expected or unexpected intermediate (mediating) and final outcomes’^116^

*Context-mechanism-outcome (CMO) configurations*: uncovered interactions between contexts and mechanisms leading to certain outcomes; providing ‘a step toward generating or refining the theory or theories that become the final product of the review’.^117^ For example, a defensive organisational culture (‘context’) leading to staff bias in recording complaints data (‘mechanism’) which results in unreliable insights for quality improvement (‘outcome’).

Previous research suggests that patients and families who make a formal complaint primarily desire two outcomes: a patient-centric response (eg, an explanation of how the incident could have happened) and system-level quality improvement (eg, to prevent errors from happening to others).^18–26^ The existing literature suggests, however, that healthcare complaints practice has not yet been successful at achieving the complex dual role of case-by-case handling and system-wide improvement.^16 17 27^ Combining organisational learning and complaint handling has previously been suggested in non-healthcare organisations, yet remains conceptual in nature, and existing literature gives little insight into how this would work in practice.^28^ To address the translational gap between developments in complaints research and current complaint handling practice, it is critical that theory to improve learning from complaints is grounded in the implementation context including an understanding of whether and how it can be linked to complaint handling practice.

Realist reviews are increasingly used in health and public services, as they recognise that the success of complex interventions is fundamentally dependent on integration into pre-existing systems, contexts and user reasoning.^29^ In contrast to systematic literature reviews that simply examine ‘what works and to what degree’, realist reviews recognise the complexity of health policy interventions, and therefore examine what it is about an intervention that works (or not), under what circumstances and why, by employing a wide range of evidence sources.^29–31^ To understand how to successfully integrate patient-centric complaint handling with quality monitoring and improvement in existing practice, this study undertook a realist review of academic literature, policy evidence and front-line insights.

## Methods

### Stage 1. Identifying the aim of the review: literature screening and lay partner codesign

The aim of this review was shaped by initial literature screening and patient lay partner involvement. Academic and policy evidence was screened to capture procedures and policy involved in healthcare complaints management and to understand patient priorities for how their complaint is handled by healthcare settings. The limited volume of complaints literature allowed for literature searching based on broad search terms (eg, ‘healthcare complaints’ or ‘patient complaints’). Searches were conducted between February 2018 and July 2019, and included academic (ie, PubMed, Google Scholar, Medline) and England-based policy searches (ie, government archives (UK), National Institute for Health and Care Excellence, Social Care Institute for Excellence, General Medical Council (UK), Care Quality Commission, Parliamentary Health and Social Care Ombudsman). Further sources were included based on reference list screening. Articles were excluded if older than 15 years, if not written in English or if they discussed informal complaints that do not require a formal response (such as online or verbal complaints). Policy sources were excluded if they were older than 5 years, did not include primary data or if they included a small sample size (eg, less than 15 interviewees or 30 complaints). A total of 216 sources were identified and screened, of which 164 sources initially met the inclusion criteria (for further review in stage 3).

To shape the review’s focus, we first conducted a rapid review of academic studies that directly explored remedies sought by complainants when they submit a complaint to healthcare settings or regulators (n=9; table 1).

**Table 1:** Summary of evidence on complainants’ main remedies sought in healthcare (stage 1)

Domain*	Complainants’ prioritisation†	Description
Quality improvement^18–26^	High	Studies consistently demonstrate that patients and public find it most important that their complaint leads to quality improvement. Complainants often seek system-level care improvement rather than an intervention in their own care.‡
A patient-centric response^18–26^	Medium-high	Of medium to high importance were outcomes related to the institution’s communication in response to the complaint, such as an explanation of how poor care could have occurred, an apology, or expression of responsibility.
Financial compensation^19 20 24–26^	Low	Most studies found that patients and public perceive financial compensation of minor importance to healthcare complaints management.
Sanctions to involved professionals 18 20 21 23 24 26	Low	Importance of sanctions to involved professionals (eg, a hard-hitting conversation or disciplinary action) was considered lowest of all outcomes, and further qualitative evidence suggests that patients and public often do not want their complaint to impact on involved staff.

*The four domains (ie, quality improvement, a patient-centric response, financial compensation and sanctions to involved healthcare professionals) or close variations thereof (eg, ‘correction’—lessons learnt, system change^25^) were consistent outcome measures identified in included studies.

†Complainants’ priority ratings were developed by the reviewers based on results of included studies that examined: relative proportion of remedy domain sought by complainants^21 23–25^ or importance ratings attributed by complainants to the various remedy domains.^18 20 22 26^

‡Only four out of nine articles^18 20 25 26^ specifically distinguished between quality improvement in their own care (eg, ‘I want a solution to my problem’^26^) and quality improvement at a systems level (eg, ‘to prevent it happening to others’^26^). All four studies indicated that complainants more frequently seek, or attribute higher scores of importance to, system-level quality improvement.

Literature screening was then discussed with patient lay partners (three participants in an initial workshop; two in each of two follow-up sessions) to determine the aim of the review and articulate key programme theories to be explored. Lay partners highlighted the accessibility of complaint procedures as an important part of complaint handling which was therefore included in the review.

### Stage 2. Defining hypothesised programme theories: expert framing and practice-based theory mapping

To develop key programme theories identified in stage 1 into hypothesised programme theories that are grounded in practice, we then interviewed 13 front-line experts at a large multisite teaching hospital in London. A topic guide was developed with questions related to the key areas of interest as identified in stage 1. Additional questions were developed to reveal activities, tools, staff and organisational context behind current practice. We conducted a purposive sampling strategy to include participants with significant exposure to complaints management at different organisational levels. Participants included: complaints manager (n=1); complaints officers (n=4); senior clinical leads responsible for monitoring complaints within their service (n=5); Patient Advice and Liaison Service manager (n=1) and officer (n=1); and quality board member (n=1). Transcripts were analysed to map current complaints processes, identify key user needs and contexts and translate our key programme theories of interest into practice-based hypothesised programme theories (table 2).

**Table 2:** Hypothesised programme theories for patient-centric complaint handling and system-wide quality improvement* (stage 2)

Procedural pathway	Programme theory title	Description
Complaint handling	Invite	Healthcare settings support and encourage patients and families to submit a complaint following negative experiences, incidents or negligence.
	Respond	Complainants receive a patient-centric response that provides an explanation of poor care, admission of responsibility and learning or action taken from their complaint.
Quality monitoring and improvement	Report	Important information from complaints is recorded in a reliable and standardised manner to allow for aggregated analysis.
	Analyse	Aggregated analysis of complaints supports the identification of systemic and severe complaints and leads to actionable insights for improvement.
	Improve	Insights derived from complaints analysis are used to inform quality improvement priorities and interventions.

*Hypothesised programme theories were conceptualised by the authors based on literature screening, lay partner involvement and 13 expert interviews.

### Stage 3. Testing hypothesised programme theories: review and synthesis of academic and policy evidence

In stage 3, hypothesised programme theories were tested and refined based on existing literature. Initially selected articles (stage 1; n=164) were appraised based on ‘theory testing potential’, that is, presence of evidence that can help explain why hypothesised programme theories might or might not work in particular circumstances, and rigour.^32 33^ Eighty-four documents were deemed ‘fit for purpose’ and were included for final analysis. In line with the realist approach, sources were assessed to develop context-mechanism-outcome (CMO) configurations.^29 34^ To extract relevant data to develop CMO configurations, a bespoke data extraction form was completed for each document. The extraction form included study design, objectives, study shortcomings and key information considered relevant to the working of one or multiple programme theories (to populate CMO configurations). Iterative analysis and synthesis of extracted data led to the final CMO configurations.^29 34^ Realist And MEta-narrative Evidence Syntheses: Evolving Standards (RAMESES) publication standards guided the reporting of the review.^33^

## Results

**Figure 1:**
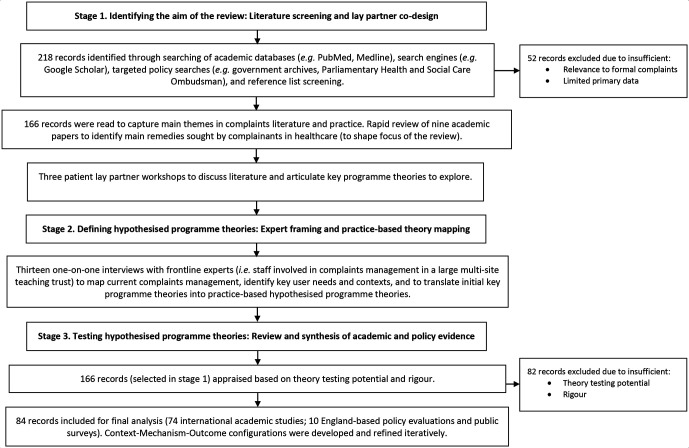
Review process and document flow.

The review process and document flow are documented in figure 1. Seventy-four academic sources were undertaken in the Netherlands (n=16), USA (n=13), UK (n=10), Sweden (n=5), New Zealand (n=5), Australia (n=4), Canada (n=4), Taiwan (n=3), Israel (n=3), France (n=2), Turkey (n=2), Denmark (n=1), Singapore (n=1), Vietnam (n=1), Italy (n=1), Japan (n=1), Norway (n=1) and Switzerland (n=1). Settings primarily included hospitals (n=47) or were conducted across multiple health services (eg, complaints submitted to national health regulators; public surveys) (n=24). Academic literature predominantly involved complaints analysis (n=49), followed by surveys or interviews of patient and public (n=11), healthcare staff (n=8), or both (n=2). Four papers were case studies of hospital complaint handling. Ten policy sources were further included that reviewed current practice in England and examined views of service users and front-line staff (case reviews, workshops, surveys).

The final programme theories are reported here and summarised in table 3. In accordance with the realist approach,^32^ reported outcomes were not necessarily main study outcomes of examined sources (eg, but relevant side findings). Although CMOs were primarily based on literature synthesis, expert interview findings guided the weighting of evidence based on relevance to implementation context.

**Table 3:** Summary of 12 context-mechanism-outcome (CMO) configurations for patient-centric complaint handling and system-wide quality improvement* (stage 3)

Procedural pathway	Relevant programme theory	Mechanism reference	Context (C)	Mechanism (M)	Outcome (O)
Complaint handling	Invite	CMO1^35–49^	Clarity of complaints procedures and policies	Patients and families are more inclined to complain if they are aware of their rights and can easily access information that outlines procedures involved.	…and facilitates patient and family access to seek redress
		CMO2^25 35–37 40–42 45 46 49–60 68^	Complainant characteristics and accompanying needs (eg, complainants burdened by health condition or language barriers)	Collaboration with support and advocacy services improves accessibility for commonly excluded patient groups.	…and increases the representativeness of complaints data
		CMO3^17 40–43 46 49 58 61–64^	Stigma of complaints and staff attitude	Staff encouragement of, and signposting to, complaint procedures reduces anxiety and stigma that prevents patients and families from filing a complaint.	…and encourages patients and families to share their feedback
	Respond	CMO4^17 21 22 24 25 38 42 48 65–67^	Staff coordination and response toolkits	Comprehensive and bespoke responding improves complainant satisfaction.	…and ensures that the complaints process provides redress
		CMO5^18–26 38 40–43 46 65 67^	National standards used to monitor the quality of complaint handling	Transparency increases accountability of complaint handling and encourages other patients and families to provide feedback.	…and encourages the use of complaints procedures
Quality monitoring and improvement	Report	CMO6^7–12 16 55 65 68–82^	Framework used to record insights held in complaints	An evidence-based reporting framework supports meaningful aggregation of complaints data.	… and leads to reliable and useful learning insights
		CMO7^10 11 16 17 80 81^	Staff type responsible for reporting, accompanying incentives and received training in complaints reporting	Standardised training and guidelines for coders who are sufficiently removed from front-line practice will increase objectivity and consistency of reporting.	… and leads to data that represent patient voice
		CMO8^16 17 48 65 71^	Informatics system used to create and retain complaints information	A centralised informatics system facilitates data monitoring and triangulation.	….and allows for effective, continuous monitoring of care issues
	Analyse	CMO9^16 52 69 75 83–92^	Frequency of complaints received at service (eg, sample size)	Conducting analysis at an appropriate organisational level enables the identification of trends of poor care.	…and helps identify system-wide care issues
		CMO10^4 5 7 10 16 69 81 93^	Staff analysis skills and data infrastructure (eg, automated dashboards, triangulation)	Combining quantitative trend analysis with targeted qualitative analysis produces granular, actionable lessons for improvement.	…and helps locate and prioritise improvement initiatives
	Improve	CMO11^7 16 17 20 26 27 38 43 45 46 48 69 70 94^	Board priorities and leadership	Board priorities and leadership shape the degree to which complaints data are used for quality monitoring and improvement.	…and allows complainants to have a greater impact on care improvement
		CMO12^16 17 36 42 43 46 60–64 87 95–98^	Organisational culture and stigma of complaints	A just culture that welcomes complaints as opportunities for learning counters negative impact of complaints on staff.	… and reduces staff apprehension towards complaints

*References included 74 international academic papers and 10 England-based policy sources.

### Invite: enabling access to and use of complaints procedures

#### CMO1: patients and families are more inclined to complain if they are aware of their rights and can easily access information that outlines procedures involved

A substantive proportion of aggrieved patients and families do not complain due to negative expectations of procedures, not knowing where to go to with their complaint, or what their rights were.^35–46^ Providing comprehensive information through a range of channels^45 47 48^ (eg, elderly patients less often access information online than younger patients^49^), outlining procedures involved, rights and potential outcomes are key in improving accessibility.

#### CMO2: collaboration with support and advocacy services improves accessibility for commonly excluded patient groups

Ethnic minority,^50–54^ lower income or education^25 36 50 51 55–57^ and, in some cases, elderly^25 50 51 57^ individuals are under-represented among complainant populations across different countries, suggesting that complaints procedures do not typically meet all user needs. Specific barriers include burden of health condition,^35 37 42^ lack of perceived power^58^ and illiteracy.^59^ Local provision of interpreting and advocacy services, and collaboration with patient and community outreach organisations, can help address such barriers and improve the representativeness of the complainant population.^40 41 45 46 49 53 60^

#### CMO3: staff encouragement of, and signposting to, complaint procedures reduces anxiety and stigma that prevents patients and families from filing a complaint

*A* prevailing stigma of complaints and negative staff attitude towards ‘complainants’^17 61–64^ were consistently reported barriers to submitting a complaint,^40–43 46^ especially in the context of longer term patient–provider care relationships.^49 58^ Some service users reported they felt more encouraged to complain if front-line staff would proactively welcome feedback and were better able to signpost to the appropriate point of contact.^40 42^

### Respond: patient-centric responding to the complainant

#### CMO4: comprehensive and bespoke responding improves complainant satisfaction

Complainant satisfaction is positively associated with a formal response that includes an admission of responsibility, an explanation of how events could have occurred and specific learning or action taken.^21 22 24 25 65–67^ This requires information from front-line staff who did not always provide comprehensive and detailed statements to the complaints team within the necessary timelines.^48 67^ Case studies report that complaint handlers are not always trained with the necessary communication skills (eg, expression of listening; empathy) to provide satisfying responses to complainants, suggesting the need for training materials and response toolkits.^17 38^

#### CMO5: transparent and accountable complaint handling encourages other patients and families to provide feedback

Although most complainants desire quality improvement^18–25^ settings often failed to inform complainants of corrective action taken following their complaint.^19 20 22 23 26 38 46 65 67^ Next to individual learning, national guidelines for healthcare settings to report, analyse and publicly share trends in complaints would strengthen accountability^42 43^ and establish a complaints process that aligns with complainant expectations (ie, systematic improvement). Demonstrable impact of complaints would also encourage more patients and families to seek redress.^40 41^

### Report: recording quality and safety issues reported in complaints

#### CMO6: an evidence-based reporting framework supports meaningful aggregation of complaints data

The rich, unstructured narrative within complaints complicates reliable and meaningful extraction of quality and safety insights.^16^ Various coding taxonomies have been developed to support complaints teams and researchers in codifying complaints reliably.^7 9 11 55 65 68–78^ To achieve reliable aggregated analysis, the taxonomy should fulfil the following minimum criteria: the categories in the framework are collectively exhaustive, mutually exclusive and reflect patient voice as reported in complaints (ie, validity).^11 79 80^ The categories should also be clear and similarly understood by different coders to support consistency (ie, inter-rater reliability)^8 12 68 69 79 81 82^ and support meaningful structuring of complaint narratives, for example, by codifying problem type, location, severity and harm reported in complaints.^10 11 80^

#### CMO7: standardised training and guidelines for coders who are sufficiently removed from front-line practice will increase objectivity and consistency of reporting

To generate reliable aggregated complaints data sets, it is essential that coders apply classification taxonomies consistently and take each complaint at face value.^80^ Although text-based coding does not involve immediate extraction of root causes in individual complaints,^16 81^ meaningful structuring of complaints data is essential to identify collective concerns of patients and families including the extent and location of systemic issues, major harm and near misses.^10^ If coding staff are sufficiently independent from front-line service and receive standardised coding guidelines and training,^11 17^ it will be more likely that national and organisational complaints data sets accurately represent patient voice (eg, rather than the care provider’s perspective).

#### CMO8: a centralised informatics system facilitates data monitoring and triangulation

Complaints are traditionally handled case by case and therefore not always included in local quality systems.^16 17^ A centralised reporting system (eg, internally linked to patient experience and incident reporting systems) can support continuous monitoring of systemic quality and safety issues^16 17 48 65 71^ and enables data triangulation for comprehensive problem analysis. A functionality to flag high-priority complaints (eg, through severity coding) could appropriately triage complaints that require immediate investigation.^65^

### Analyse: deriving actionable and system-wide learning insights

#### CMO9: conducting analysis at an appropriate organisational level enables the identification of trends of poor care

A sufficiently large sample of complaints is required for aggregated analysis to detect meaningful trends of problematic care.^16 69^ Depending on the frequency of complaints at a particular healthcare setting, complaints data can either support the identification of under-recognised areas of poor practice or function as a secondary source of granular data to better understand acknowledged quality and safety issues from the patient perspective. However, even for small healthcare settings, it is critical that reliable coding outputs are produced locally and shared externally to enable national monitoring of complaints. Quantitative data outputs should however not be used independently to measure or benchmark between-setting care performance as the risk of receiving complaints is not evenly distributed across clinicians, specialties, procedural risks and patient characteristics.^52 75 83–92^

#### CMO10: combining quantitative trend analysis with targeted qualitative analysis produces granular, actionable lessons for improvement

Quantitative complaints analysis studies highlight the need for additional qualitative analysis to derive granular and actionable learning lessons.^7 69 81^ A two-step ‘spotlight’ approach has been suggested that combines quantitative trend analysis with targeted qualitative analysis.^10^ If coding is performed in a meaningful and consistent manner, quantitative complaints trends can identify, for example, the extent and location of harm, near misses and blind spots (eg, admission or discharge, systemic and omission problems) at a national and organisational level. By locating systemic issues reported across complaints, healthcare settings are then able to zoom in to areas of unsafe care and perform deeper qualitative investigation to identify contextual causes and human factors that allow for common error. The potential of further triangulation with patient feedback and incident reporting systems has been recognised^5 16^ although overlap appears somewhat inconsistent.^4 5 93^

### Improve: translating complaints insights into quality improvement

#### CMO11: board priorities and leadership shape the degree to which complaints data are used for quality improvement

At present, there is little evidence of systematic use of complaints data for system-wide problem resolution—with improvements being limited to local issues.^16 17 27^ Complainants^7 20 70^ perceive social and institutional issues as critical aspects of care quality. Yet, non-clinical complaints are unlikely to be prioritised by care providers and regulators.^20 26 69 94^ If complaints are strictly secondary to internal quality and safety data sets, they may not reveal the issues that are critical to patients but not to staff. Leadership commitment to perceive complaints as a valuable, independent data set for improvement is necessary to increase their impact.^17 38 43 45 46 48^

#### CMO12: a just culture that welcomes complaints as opportunities for learning counters negative impact of complaints on staff

Due to prevailing stigma, complaints still impact negatively on staff well-being and are often perceived as threatening or unwarranted.^42 61–64 95–98^ A just culture may relieve negative impact of complaints on staff well-being and enhance openness to learning.^16 17 36 43 46 60–62^ Accordingly, system-wide complaints analysis—in contrast to using complaints to predict individual clinician risk (eg, Predicted Risk Of New Event (PRONE) scores^87^)—facilitates focus on structural causes that allow for recurring harm (rather than individual blame).

### Situating quality monitoring and improvement in existing complaint handling practice

Exploring our CMOs in the context of existing practice in a large multisite teaching hospital (ie, 13 expert interviews) revealed unrecognised tensions between traditional case-by-case complaint handling and system-wide quality monitoring and improvement.

First, complaints did not always reach the complaints department as patients and front-line staff were not always aware of the difference between formal and informal complaints. Informal complaints were higher in number but not officially reported on. Formal complaints were classified and publicly shared following the national reporting framework.^2^ However, in practice, the immediacy of resolving a complaint took precedence over coding, as the taxonomy was not perceived to generate meaningful information (but rather, a tick box exercise). Subsequent analysis of reported data was considered a time-consuming process, including manual processing of data, requiring skills and expertise beyond the role of a complaints manager. Although the complaints informatics system was integrated with patient experience data, identification of systemic complaints and triangulation with wider patient feedback reports relied on memory and word of mouth—complicating identification of under-recognised or system-wide issues. The primary role of the complaints department was to investigate individual complaints and decide whether a complaint would be considered ‘upheld’. Although this occasionally led to individual improvements, these were largely localised, one-by-one solutions.

**Figure 2:**
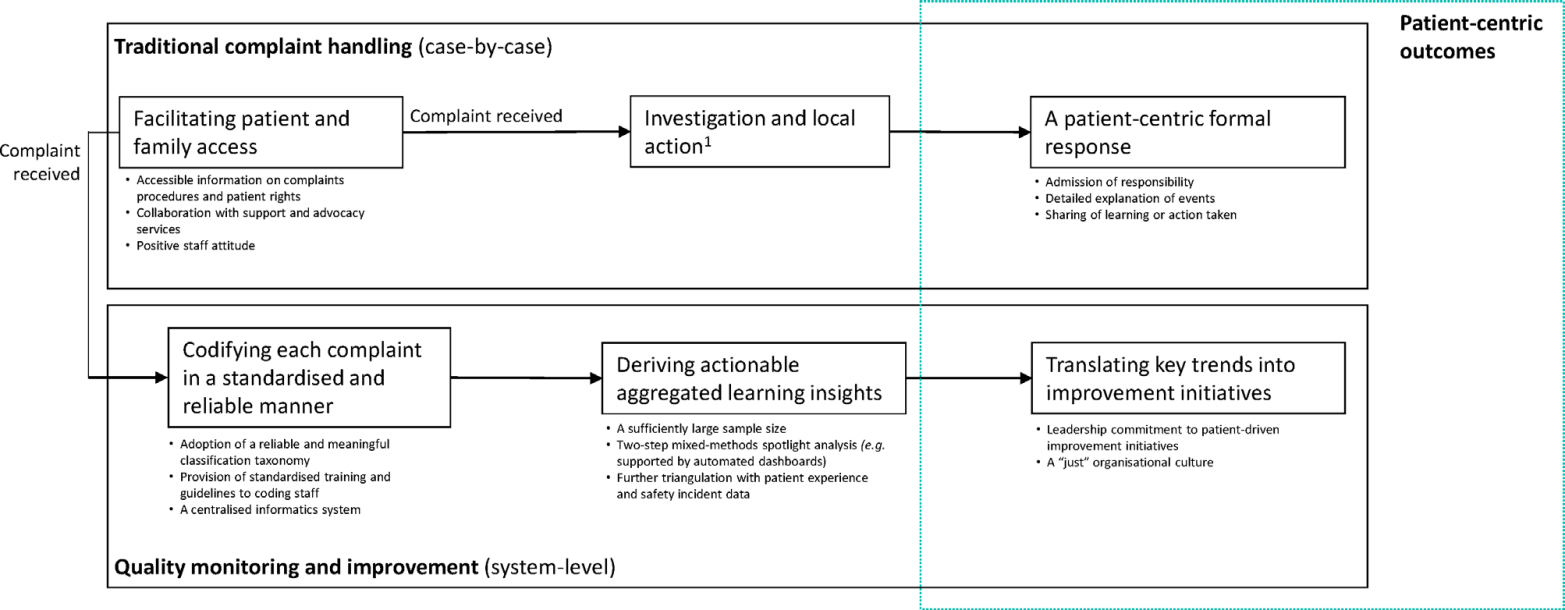
Mechanisms for patient-centric complaint handling and system-wide quality improvement. ^1^This step was not included in the review due to limited available literature.

These findings highlighted the need for better policy, tools and guidance to establish a quality monitoring and improvement pathway that is distinct from immediate, case-by-case practice. Our literature review suggests a more meaningful complaints taxonomy and guidelines (CMO6, CMO7); an effective analysis strategy to identify key hotspots and blind spots (eg, automated dashboards or analysed by healthcare informatics staff) (CMO9, CMO10); information infrastructure that allows for further data triangulation (CMO8); and leadership commitment to using complaints data to trigger and prioritise patient-driven improvement initiatives (CMO11, CMO12) (figure 2). At the case-by-case level, improving access to formal complaints (eg, better patient information and staff education) (CMO1–3) and patient-centric responding to specific concerns raised (CMO4–5) will further remain imperative to securing patient and family redress.

## Discussion

This review involved patients and front-line experts, and reviewed academic and policy evidence, to understand how to effectively integrate patient-centric complaint handling with quality monitoring and improvement. In complaints literature, the complex reality of a dual objective system has not been adequately addressed. Complaints literature could largely be divided into two fields. First, studies that examined aspects of complaint handling (eg, complainant expectations or clinician experience of receiving a complaint). Second, studies that analyse complaints data to support quality improvement (eg, identifying recurring problem themes in complaints). Complaint handling literature indicates that system-level improvement is an essential outcome for complainants in healthcare,^18 20 25 26^ yet did not address how to process and use complaints to achieve this. Complaints analysis studies have generated promising methodologies to unlock the value of complaints, yet were rarely situated in practice. It is therefore somewhat unsurprising that policy evidence and expert insights echo earlier studies^16 17 27^ that suggest improvement initiatives do not often move beyond ‘putting out fires’.^27^

Our review contributes to the existing literature by providing pragmatic insights on how, why and under what conditions complaints can be systematically learnt from in existing practice. Our review suggests that, although complaints necessarily require case-by-case handling, there is a need for novel policy strategies that enable a distinct improvement pathway to address systemic and system-wide issues reported in complaints. If healthcare settings are better supported to codify, analyse and use complaints data (eg, through standardised taxonomy and guidelines), patient-reported insights could impact quality management in important ways. First, meaningful structuring of complaints data (eg, filtering complaints through ‘severity’ coding^11 80^) could support effective triage of critical patient concerns through the appropriate safety management processes. For example, blind spot issues held in complaints (eg, preadmission, postdischarge or systemic issues^10^) may be used to trigger deeper investigation into critical incidents that are under-reported by staff^99^ (eg, near misses^100^ or incidents that occur over time^101^). Second, the complexity and granularity of complaints data mean it can function as a secondary data source to better understand quality or safety issues exposed by other feedback and incident reporting systems.^5^ Patient-reported narratives tend to describe the patient’s journey across care visits and settings, including social and institutional events before and after patient harm.^102–104^ This could help address some of the known issues with root cause analysis,^105 106^ such as the limited value of internal incident data (eg, fragmented and clinically focused). Similarly, complaints could be linked to overall patient satisfaction rates to reveal latent incidents that may explain changes over time.^91 107^ Most importantly, ensuring reliability and validity of national and institutional-level complaints data sets will be imperative to unlocking the collective voice of complainants. Reliable complaints data sets underpin the function of complaints as a public accountability mechanism to govern care quality, safety, and patient-centricity. By revealing systemic patient concerns (including low-severity but frequently reported care issues), complaints could support the development and prioritisation of patient-centric improvement initiatives (which could include further patient codesign^108–110^).

Although improved analysis of complaints allows patients and families to have a greater impact on health systems, it is important to note that complaints data are unlikely to be representative of the overall patient population. Our findings reinforce work to address poor accessibility of complaints procedures,^40–43^ ongoing stigma of complaints^46 60 61 63^ and a defensive organisational culture.^17 43 46 60^ Without accessible and equitable complaints procedures, complaints data may only represent the ‘tip of the iceberg’ and disproportionately omit learning from ethnic minority^50–54^ elderly^25 50 51 57^ and lower income or education populations.^25 36 50 51 55–57^ It is therefore important to understand complaints data in the context of other patient voice mechanisms (eg, satisfaction surveys, public consultations). An essential first step to effective triangulation of different data sources is to understand how to meaningfully structure and analyse each data set individually. It can be expected that some of the findings in this review (eg, standardisation of coding, spotlight analysis) apply to the processing of other free-text feedback mechanisms (eg, informal complaints, online comments).

### Study strengths and limitations

In line with the realist review approach, this paper has reviewed heterogeneous evidence sources (eg, expert interviews, academic literature, public consultations) allowing for a nuanced understanding of all aspects of complaints management and policy.^111^ Although this is an important strength of the review, it somewhat limited our ability to establish saturation in some of the review’s findings. A limited body of evidence further meant that our CMOs are by no means exhaustive and do not necessarily include all processes involved in complaints management (eg, there was insufficient evidence on investigative procedures involved in complaint handling). Furthermore, most of the selection, extraction and appraisal of literature was conducted independently by a single researcher (JD) leading to potential bias.^111–113^ Measures were taken accordingly to maximise standardisation (eg, data extraction form, rigid appraisal criteria). Finally, although a large proportion of the evidence (n=74) was drawn from a range of countries, policy sources (n=10) and expert interviews were based on NHS practice in England, and often secondary care. Some of the reported issues and contexts of existing practice may therefore not directly translate to other settings.

## Conclusion

Informed by evidence on complainant priorities and lay partner codesign, this study has conducted a realist review of academic and policy research to understand how to effectively integrate patient-centric complaint handling with quality monitoring and improvement. Thirteen front-line experts from a large multisite hospital were involved to ensure theory for change would be relevant to practice and achievable in short term. Our findings highlight the need to develop novel policy strategies that sufficiently distinguish complaints reporting, analysis and improvement from complaint handling practice, and include findings on who is best placed for reporting and analysis (eg, independent staff, analysis skills), the necessary tools and training (eg, reliable, valid and useful reporting framework), an analysis strategy to generate actionable learning insights (eg, mixed-methods ‘spotlight’ approach) and translation into quality improvement (eg, leadership and culture). This is critical for patients and families, who aim to drive quality improvement, and for healthcare providers, who could learn from their experiences to provide safer, more patient-centric care.
